# Supplementary material for: A human-based multi-gene signature enables quantitative drug repurposing for metabolic disease
Source: eLife. 2022 Jan 17;11:e68832. doi: 10.7554/eLife.68832 (PMC8763401; doi:10.7554/eLife.68832)
Supplement: Source code 1. [file elife-68832-supp2.zip › Source_code_file_R1.docx]

**Terminal, R and Python Code**

**Example pre-processing and normalisation (using terminal on OS)**

FIND MEDIAN

./findmedian -c hta20_Hs_ENST_19,binary.cdf *.CEL > findmedian.txt

REPORT MEDIAN

tail -2 findmedian.txt | head -1 | cut -f 4

cat findmedian.txt | grep ‘^Median CEL:’ | cut -f 4

RUN IRON

./iron -c hta20_Hs_ENST_19,binary.cdf --norm-iron=MEDIAN.CEL *.CEL -o iron_output.txt

**#lm with the output wrapped by anova() and so a Type I output & plots the data # See https://www.rdocumentation.org/packages/stats/versions/3.6.2/topics/lm**

rm(list=ls())

library(data.table)

library(dplyr)

library(multtest)

## samples in columns and genes as rows

data <- read.table("IRON_processed_expression_data_log2.csv",sep=",",header=TRUE,row.names = 1, check.names=FALSE)

head(data[1:5,1:5])

#select the correct samples

# ID_index <- as.matrix(read.table("SMP-192_sample_list.txt", sep="\t", header=FALSE))

# idx <- match(as.character(ID_index[,1]),rownames(data))

# head(idx)

# data <- t(data[idx,])

ps_index <- read.table("Subset_of_common_ENST_across_array_types.csv", sep=",", header=TRUE)

psx <- match(as.character(ps_index[,1]),rownames(data))

Subsetdata <- data[psx,]

data <- as.matrix(Subsetdata)

filenameUni <- 'IRON_processed_expression_data_53k_ENST_Age_x_HOMA2IR_unimodal'

filenameUniPDF <- 'IRON_processed_expression_data_53k_ENST_Age_x_HOMA2IR_unimodal.pdf'

insulinAnot <- as.matrix(read.table("Age_log_HOMA2_phenotype.csv", sep=",", header=TRUE, row.names=1))

### DATA AND ANOTATION MUST BE IDENTICAL INCLUDING ORDER

mpOnly <- grep(rownames(insulinAnot), pattern="STUDY") ### Change "STUDY" study ID change here

insulinAnot <- insulinAnot[mpOnly,] ## Selects samples

data <- data[,rownames(insulinAnot)] ## ensures data contains the same samples and same order as phenotype file

############################

## model used for the multivariate analysis

myModel <- "Age*HOMA2IR"

##This is a function to adjust P values

multCor <- function(pVec, corMethod="BH"){

library(multtest)

tmpAdj <- mt.rawp2adjp(as.vector(pVec), proc=corMethod)

returnObj <- as.vector(tmpAdj$adjp[order(tmpAdj$index),2])

return(returnObj)

}

## first univariate analysis and plot unimodal data.

plotUni <- function(inData=NULL, pdfName=filenameUniPDF, insulinData=NULL){

myNames <- rownames(inData)

pdf(pdfName, width=12, height=12, pointsize=1/300)

par(mfrow=c(3,2), cex=2)

myOutStats <- c()

for(tmpId in myNames){

##check anotation and data follows the same order

insulinData <- insulinData[colnames(inData),]

plot(x=inData[tmpId,], y=insulinData[,"Age"], pch=16, ylab="AGE", xlab="Gene expression", main=tmpId)

##run regressions for all data

lm <- lm(insulinData[,"Age"]~inData[tmpId,])

lmanova <- anova(lm)

##get positions for lines and plot these

minX <- min(inData[tmpId,])

maxX <- max(inData[tmpId,])

minY <- lm$coefficients[1] + (minX*lm$coefficients[2])

maxY <- lm$coefficients[1] + (maxX*lm$coefficients[2])

lines(x=c(minX, maxX), y=c(minY, maxY), col=1, lwd=2)

c2R <- cor( inData[tmpId, ], insulinData[,"Age"], use="pairwise.complete.obs")

coefDet <- cor( inData[tmpId, ], insulinData[,"Age"], use="pairwise.complete.obs")^2

c2Slope <- lm$coefficients[2]

##print stats

legend("top", cex=0.6, c(paste0("lm p: ", signif(lmanova[1,5], digits=2)), paste0("R2: ", signif(coefDet, digits=2))))

tmpStats <- c(lmanova[1,5], c2R, coefDet, c2Slope)

## Next HOMA2IR

plot(x=inData[tmpId,], y=insulinData[,"HOMA2IR"], pch=16, ylab="HOMA2IR", xlab="Gene expression", main=tmpId)

##run regressions for all data

lm <- lm(insulinData[,"HOMA2IR"]~inData[tmpId,])

lmanova <- anova(lm)

##get positions for lines and plot these

minX <- min(inData[tmpId,])

maxX <- max(inData[tmpId,])

minY <- lm$coefficients[1] + (minX*lm$coefficients[2])

maxY <- lm$coefficients[1] + (maxX*lm$coefficients[2])

lines(x=c(minX, maxX), y=c(minY, maxY), col=1, lwd=2)

c2R <- cor( inData[tmpId, ], insulinData[,"HOMA2IR"], use="pairwise.complete.obs")

coefDet <- cor( inData[tmpId, ], insulinData[,"HOMA2IR"], use="pairwise.complete.obs")^2

c2Slope <- lm$coefficients[2]

##print stats

legend("top", cex=0.6, c(paste0("lm p: ", signif(lmanova[1,5], digits=2)), paste0("R2: ", signif(coefDet, digits=2))))

##collect stats

tmpStats <- c(tmpStats, lmanova[1,5], c2R, coefDet, c2Slope)

#############################

myOutStats <- rbind(myOutStats, c(tmpStats))

}

dev.off()

rownames(myOutStats) <- rownames(inData)

colnames(myOutStats) <- c("Age_p", "Age_R", "Age_R2", "Age_slope", "HOMA2IR_p", "HOMA2IR_R", "HOMA2IR_R2", "HOMA2IR_slope")

##Add fdrs

ageFdr <- multCor(myOutStats[,"Age_p"])

HOMA2IRFdr <- multCor(myOutStats[,"HOMA2IR_p"])

myOutStats <- cbind(myOutStats[,"Age_p"], ageFdr, myOutStats[,c("Age_R", "Age_R2", "Age_slope")], myOutStats[,"HOMA2IR_p"], HOMA2IRFdr, myOutStats[,c("HOMA2IR_R", "HOMA2IR_R2", "HOMA2IR_slope")])

colnames(myOutStats) <- c("Age_p", "Age_fdr", "Age_R", "Age_R2", "Age_slope", "HOMA2IR_p", "HOMA2IR_fdr", "HOMA2IR_R", "HOMA2IR_R2", "HOMA2IR_slope")

return (myOutStats)

}

########## multivariate analysis

unimodalMulti <- function(inData=NULL, insulinData=NULL, formula=myModel){

myNames <- rownames(inData)

statsCollect <- c()

coefCollect <- c()

R2Collect <- c()

for(tmpId in myNames){

tmpData <- cbind(inData[tmpId,], insulinData[,])

##need to remove data with NAs for any of the parameters...

naSum <- apply(is.na(tmpData), 1, sum)

tmpData <- tmpData[naSum==0,]

colnames(tmpData)[1] <- "exp"

modFormula <- paste0("exp~", formula)

tmpData <- as.data.frame(tmpData)

attach(tmpData)

tmpDataLm <- lm(as.formula(modFormula))

detach(tmpData)

tmpDataLmanova <- anova(tmpDataLm)

statsCollect <- rbind(statsCollect, tmpDataLmanova[,5])

coefCollect <- rbind(coefCollect, tmpDataLm$coefficients)

##Calculate R2 from sums of squares

##Will do this stepwise to continuously reduce the total sums of squares by the part already accounted for

totalSoS <- sum(tmpDataLmanova[,2])

tmpR2 <- c()

for(row in 1:c(nrow(tmpDataLmanova)-1)){

tmpR2 <- c(tmpR2,tmpDataLmanova[row,2]/totalSoS)

##This is the stepwise reduction of the SoS...

totalSoS <- totalSoS - tmpDataLmanova[row,2]

}

R2Collect <- rbind(R2Collect, c(tmpR2, NA))

}

colnames(statsCollect) <- rownames(tmpDataLmanova)

colnames(coefCollect) <- names(tmpDataLm$coefficients)

rownames(statsCollect) <- rownames(coefCollect) <- myNames

statsCollectFdr <- apply(statsCollect, 2, multCor)

rownames(statsCollectFdr) <- rownames(statsCollect)

## get the Rs

RCollect <- sqrt(R2Collect)

RCollect[,1:c(ncol(RCollect)-1)] <- RCollect[,1:c(ncol(RCollect)-1)]* sign(coefCollect[,2:ncol(coefCollect)]) ##Tricky need to exclude the intercept and aviod the resid column

rownames(R2Collect) <- rownames(RCollect) <- rownames(statsCollect)

colnames(R2Collect) <- colnames(RCollect) <- colnames(statsCollect)

outList <- list("statsP"=statsCollect,

"statsFDR"=statsCollectFdr,

"coefs"=coefCollect,

"R"=RCollect,

"R2"=R2Collect)

return(outList)

}

##############################################################################Unimodal analysis

myUnimodalUnivariate <- plotUni(inData=data, insulinData=insulinAnot)

myUnimodalMultiVarOut <- unimodalMulti(inData=data, insulinData=insulinAnot)

unimodalMultiStatsP <- myUnimodalMultiVarOut$statsP

unimodalMultiStatsFDR <- myUnimodalMultiVarOut$statsFDR

uniModalMultiCoefs <- myUnimodalMultiVarOut$coefs

uniModalMultiR <- myUnimodalMultiVarOut$R

############################################## Write output tables for unimodal analysis

## univariate output

write.table(myUnimodalUnivariate, file=paste0(filenameUni, "_Unistats.txt"), sep="\t", quote=T, row.names = TRUE ,col.names =TRUE)

## multivariate outputs

write.table(unimodalMultiStatsP, file=paste0(filenameUni, "_multiStatsP.txt"), sep="\t", quote=T, row.names = TRUE ,col.names =TRUE)

write.table(uniModalMultiCoefs, file=paste0(filenameUni, "_multiCoefs.txt"), sep="\t", quote=T, row.names = TRUE ,col.names =TRUE)

write.table(uniModalMultiR, file=paste0(filenameUni, "_multiR.txt"), sep="\t", quote=T, row.names = TRUE ,col.names =TRUE)

write.table(unimodalMultiStatsFDR, file=paste0(filenameUni, "_multiStatsFDR.txt"), sep="\t", quote=T, row.names = TRUE ,col.names =TRUE)

**# Meta-analysis of pvaules using MetaDE.pvalue ----------------------------#**

library(MetaPath)

library(GSA)

library(Biobase)

library(genefilter)

library(GSEABase)

library(limma)

library(MetaDE)

rm(list=ls())

x <- read.table("3M_n_D_n_H_delta_mP.csv", sep=",", header=TRUE, row.names=1)

x_list <- list(p=x)

x.analysis <- MetaDE.pvalue(x_list, meta.method = c("Stouffer"), miss.tol = 0, asymptotic=F)

# extract the p values for the meta analysis from list object

metA_pvals <- x.analysis$meta.analysis$pval

# extract FDR for meta analysis

metA_FDR <- x.analysis$meta.analysis$FDR

# are gene ids in same order

all.equal(rownames(x), rownames(metA_pvals), rownames(metA_FDR))

# bind to original data

meta_p_fdr_data <- cbind(x, metA_pvals, metA_FDR)

# change last colname to something sensible

colnames(meta_p_fdr_data)[length(meta_p_fdr_data)] <- 'FDR'

filename='3M_n_D_n_H_delta_mP_Stouffer'

write.table(meta_p_fdr_data, file=paste0(filename, "_.csv"), sep=",", quote=T, row.names = TRUE ,col.names =TRUE)

Excel and simple functions are then used to identify consistent directionality for CC values.

**DeepPurpose implementation (Python).**

# Prerequisite: DeepPurpose

# https://github.com/kexinhuang12345/DeepPurpose.git

from DeepPurpose import DTI as models

import warnings

import pandas as pd

from sklearn.preprocessing import MinMaxScaler

# Available pretrained models

pretrained_models = ['CNN_CNN_DAVIS',

'CNN_CNN_BindingDB',

'Morgan_CNN_BindingDB',

'Morgan_CNN_DAVIS',

'Morgan_AAC_KIBA',

'MPNN_CNN_BindingDB',

'MPNN_CNN_KIBA',

'MPNN_CNN_DAVIS',

'Transformer_CNN_BindingDB',

'Daylight_AAC_DAVIS',

'Daylight_AAC_KIBA',

'Daylight_AAC_BindingDB',

'Morgan_AAC_BindingDB',

'Morgan_AAC_DAVIS']

# Inference set

test = pd.read_csv('inference_set_EGFR.csv')

t = test['sequence'].values

d = test['SMILES'].values

# Run pretrained models DeepPurpose

for pretrained_model in pretrained_models:

print('###STARTING MODEL####:', pretrained_model)

model = models.model_pretrained(model=pretrained_model) # Initialise model

y_pred = models.virtual_screening(d, t, model) # VS

test[pretrained_model] = y_pred

scaler = MinMaxScaler() # Scale prediction 0-1 range

scaled_pred = pretrained_model + '_scaled'

test[scaled_pred] = scaler.fit_transform(test[[pretrained_model]])

# Write predictions

test.drop(columns=['sequence', 'SMILES']).to_csv('inference_set_EGFR_out.csv', index=False)

**Calculate rank fusion scores**

import pandas as pd

import numpy as np

def rank_fusion(input_df):

'''Calculate rank fusion

by mean ranking across models'''

df = input_df.copy()

cols = [model + '_rank' for model in pretrained_models]

df['mean_rank'] = df[cols].mean(axis=1)

df = df.sort_values(by='mean_rank')

df['rank_fusion'] = np.array(range(len(df))) + 1

cols_output = ['name', 'gene', 'rank_fusion'] + cols

df = df[cols_output]

return df

# DeepPurpose output

df = pd.read_csv('inference_set_EGFR_out.csv')

pretrained_models = ['CNN_CNN_DAVIS',

'CNN_CNN_BindingDB',

'Morgan_CNN_BindingDB',

'Morgan_CNN_DAVIS',

'Morgan_AAC_KIBA',

'MPNN_CNN_BindingDB',

'MPNN_CNN_KIBA',

'MPNN_CNN_DAVIS',

'Transformer_CNN_BindingDB',

'Daylight_AAC_DAVIS',

'Daylight_AAC_KIBA',

'Daylight_AAC_BindingDB',

'Morgan_AAC_BindingDB',

'Morgan_AAC_DAVIS']

# Unique compounds

cpds = df['name'].drop_duplicates()

# For each compound compute rank fusion and write output file

for cpd in cpds:

cpd_df = df[df['name'] == cpd]

rank_array = np.array(range(len(cpd_df))) + 1

for model in pretrained_models:

cpd_df = cpd_df.sort_values(by=model, ascending=False)

col_name = model + '_rank'

cpd_df[col_name] = rank_array

cpd_df = rank_fusion(cpd_df)

file_name = cpd + '_rankings.csv'

cpd_df.to_csv(file_name, index=False)
